# A tool for mapping Single Nucleotide Polymorphisms using Graphics Processing Units

**DOI:** 10.1186/1471-2105-15-S1-S10

**Published:** 2014-01-10

**Authors:** Andrea Manconi, Alessandro Orro, Emanuele Manca, Giuliano Armano, Luciano Milanesi

**Affiliations:** 1 National Research Council, Institute for Biomedical Technologies, Segrate (MI), 20090, Italy; 2 Department of Electrical and Electronic Engineering, University of Cagliari, Cagliari (CA), 09123, Italy

## Abstract

**Background:**

Single Nucleotide Polymorphism (SNP) genotyping analysis is very susceptible to SNPs chromosomal position errors. As it is known, SNPs mapping data are provided along the SNP arrays without any necessary information to assess in advance their accuracy. Moreover, these mapping data are related to a given build of a genome and need to be updated when a new build is available. As a consequence, researchers often plan to remap SNPs with the aim to obtain more up-to-date SNPs chromosomal positions. In this work, we present G-SNPM a GPU (Graphics Processing Unit) based tool to map SNPs on a genome.

**Methods:**

G-SNPM is a tool that maps a short sequence representative of a SNP against a reference DNA sequence in order to find the physical position of the SNP in that sequence. In G-SNPM each SNP is mapped on its related chromosome by means of an automatic three-stage pipeline. In the first stage, G-SNPM uses the GPU-based short-read mapping tool SOAP3-dp to parallel align on a reference chromosome its related sequences representative of a SNP. In the second stage G-SNPM uses another short-read mapping tool to remap the sequences unaligned or ambiguously aligned by SOAP3-dp (in this stage SHRiMP2 is used, which exploits specialized vector computing hardware to speed-up the dynamic programming algorithm of Smith-Waterman). In the last stage, G-SNPM analyzes the alignments obtained by SOAP3-dp and SHRiMP2 to identify the absolute position of each SNP.

**Results and conclusions:**

To assess G-SNPM, we used it to remap the SNPs of some commercial chips. Experimental results shown that G-SNPM has been able to remap without ambiguity almost all SNPs. Based on modern GPUs, G-SNPM provides fast mappings without worsening the accuracy of the results. G-SNPM can be used to deal with specialized Genome Wide Association Studies (GWAS), as well as in annotation tasks that require to update the SNP mapping probes.

## Background

GWAS have shown that genetic variants are often responsible of traits expressed in phenotypes. Genetic variants may be associated with the cause (e.g., [1]) or with the predisposition (e.g., [2]) of a disease, and may determine individual drug responses (e.g., [3]). SNPs are the most common type of genetic variant in human genome. More than 10 million SNPs are estimated to be in the human genome [4]. The scientific community has placed a great interest in the analysis of SNPs, widely exploiting their knowledge in GWAS [5-7]. Hence, different public resources have been devised to share their knowledge (e.g., dbSNP [8], the International HapMap Project [9], the 1000 Genomes Project [10]), as well as specialized tools for SNP calling (e.g. MAQ [11], SOAPsnp [12], SNVMix [13]) and SNP analysis (e.g., FAST-SNP [14], SNPLims [15], SNPInfo [16], SNPranker 2.0 [17]). In this context, SNP genotyping arrays represent an important tool for genetic analysis. It should be pointed out that the reliability of the genotype-phenotype associations that may be discovered analyzing SNPs is strongly related to the accuracy of the data that describe them. In particular, SNP genotyping analysis is very susceptible to SNPs chromosomal position annotation errors. In fact, wrongly mapped SNPs may in some cases affect data analysis and lead to erroneous conclusions. An interesting study about wrongly mapped SNPs in commercial SNP chips, and on their possible functional consequences, has been presented in [18]. In this work, SNPs of various chips have been remapped using highly sensitive alignment parameters against their reference genomes, with the goal to highlight discrepancies between the found genomic positions and those provided by the chip vendors. These discrepancies highlighted that more sensitive aligner parameters should be used to achieve an accurate alignment instead of retrieving a partial best alignment with extra SNPs, indels or less SNP flanking sequence aligned. This suggests that researchers should closely examine how mapping data have been obtained, with the goal of analyzing their accuracy and if necessary taking into account the opportunity to update them. However, mapping data are provided to the users along the SNP chips, omitting any information about the algorithm and the parameter settings used to obtain them. Then, meticulous researchers often plan to remap the SNPs to obtain more accurate chromosomal positions before performing association studies. In general, when a new build of a genome is available it might be productive to re-analyze the data of old genotyping experiments while exploiting the new reference sequences. In this case, as the mapping data of SNP chips are related to a given build of the genome under consideration (irrespective of their original accuracy), chromosomal positions need to be updated according to the newest build. Moreover, in genotyping analysis often researchers need to merge genetic datasets coming from different genotyping platforms, which in turn use different sets of SNPs to represent genetic polymorphisms. To this end, it is necessary to know the exact position of a SNP in a chromosome and update this information when new builds of the reference genome are available.

Specialized tools as LiftOver [19], AssemblyConverter [20], and the NCBI Genome Remapping Service [21] have been devised to project the coordinates of genomic regions from a given build to another build of a genome. These tools are very useful to update chromosomal coordinates between different reference sequences; however they might be unable to perform a given conversion between different assemblies. In fact, these tools typically allow only a limited set of assembly-assembly conversion combinations. Then, it might be impossible to use them to update SNPs positions on a given build of a genome. Moreover, new positions obtained using these tools are strongly related to the initial positions provided by the vendor. Unfortunately, if a SNP has been previously wrongly mapped by the vendor, the error will be spread to the updated position. Finally, these tools are specialized to convert coordinates from a build to another and do not permit to remap a SNP against the same reference build to look for discrepancies with the vendor positions.

Researchers use tools as BLAST [22] or BLAT [23] to analyze the SNP probes positions and/or to update them to the genome or to the transcriptome. For instance, some researchers highlighted that many of the Illumina probes have unreliable original annotations and defined a pipeline that exploits both BLAST and BLAT to perform complete genomic and transcriptomic re-annotation of the probe sequences [24]. AffyProbeMiner [25] is a platform-independent tool that uses all RefSeq mature RNA protein coding transcripts and validated complete coding sequences in GenBank [26] to regroup the individual probes into consistent probe sets to remap them to the correct sets of mRNA transcripts exploiting a local implementation of the BLAT server. The Bioconductor [27] package named *altcdfenvs *has been used to investigate how probes found on Affymetrix microarrays were matching on more recent curated collections of human transcripts. Experiments showed that not all the probes matching a reference sequence were consistent with the grouping of probes by the manufacturer of the chips [28]. However, using tools as BLAST or BLAT to update thousands or millions of SNPs is a very expensive task in terms of computing time.

In this work, we present an improved version of G-SNPM (standing for GPU-SNP Mapping) [29], an accurate and very fast tool devised to cope with the problem of updating SNPs chromosomal positions. Written in Python, G-SNPM is mainly based on the SOAP3-dp [30] short-read mapping tool to exploit the computation power of modern GPUs.

G-SNPM is available at the following address http://www.interomics.eu/sp1-wp2.

## Methods

G-SNPM is a tool that maps a sequence representative of a SNP against a reference sequence in order to find the absolute position of the SNP in that sequence. For genotyping analysis a SNP is represented by a oligonucleotide probe for each possible allele. In turn, these probes can be synthetically described by a regular expression obtained by combining the flanking sequences of a SNP with a grouping construct that represents its possible alleles (e.g., GCACTCTCACATGGATTAGGGAATTA[CG]ATGCAGACCTCCTGCACAACTGCCC). Since public repositories as dbSNP provide short and fixed length flanking sequences, we assume that typically the probes used to design a SNP chip are represented by short sequences. Starting from this consideration, a short-read mapping tool could be successfully used to cope with the SNP mapping task.

In the following of this section, we first introduce existing state-of-the-art short-read mapping tools. Then, we propose our strategy, devised to deal with SNP mapping problems. Successively, we discuss about the adopted alignment constraints. Finally, we briefly resume the minimal hardware and software equipment required to use G-SNPM.

### Short-read mapping tools

Several tools have been devised to perform short-read mappings. Without aiming to be exhaustive, let us cite some of the most popular solutions, as MAQ [31], RMAP [32,33], Bowtie [34], BWA [35], CloudBurst [36], and SHRiMP2 [37,38]. MAQ maps short sequence reads to a reference genome by calculating the probability of a read alignment to be correct, and consensus genotype calling with a model that incorporates correlated errors and diploid sampling. It supports gapped alignment and can align reads up to 128 bp. RMAP uses quality scores to provide accurate ungapped alignments. In so doing, it exploits two different mapping criteria. A first criterion is based on a simple count of mismatches between a read and the aligned genomic region, while a second criterion makes use of the base-call quality scores. By manipulating the quality-score cutoff, the second criterion provides another means of adjusting sensitivity and specificity. In particular, it allows positions to contribute when they are of high-quality, but not be penalizing if they are low-quality. Bowtie is a memory-efficient short-read aligner that exploits the Burrows-Wheeler Transform (BWT) to index the genome allowing only ungapped alignments. BWA is another tool that exploits the BWT to index the reference sequences. It can also provide gapped alignments, while Bowtie cannot. It consists of three algorithms (i.e., BWA-backtrack, BWA-SW and BWA-MEM), devised to perform both short and long read alignments. CloudBurst is a parallel seed-and-extend read-mapping tool able to align reads with a specified number of differences, including both mismatches and indels (insertions/deletions). It exploits the open-source Hadoop [39] implementation of MapReduce [40] to parallelize the execution using multiple computing nodes. SHRiMP2 exploits specialized vector computing hardware to speed-up the Smith-Waterman [41] dynamic programming algorithm. It is a multi-core short-read mapping tool that enables the alignment of reads with extensive polymorphism and sequencing errors. A comparative study aimed at assessing the accuracy and the runtime performance of different state-of-the-art Next-Generation Sequencing (NGS) read alignment tools highlighted that among all SOAP2 [42] is the one that showed the higher accuracy [43]. Exhaustive reviews of the tools cited above can be found in the literature (e.g., [44]).

In general, the mentioned solutions exploit some heuristics to find a good compromise between accuracy and running time. Recently, GPU-based solutions have been proposed to cope with different bioinformatics problems [45-48]. GPUs have also been exploited to cope with the exponentially increasing throughput of NGS. In particular, the computational power of these hardware accelerators is helping researchers to speed the short-read mapping process without compromising accuracy and sensitivity. Lately, the GPU-based short-read mapping tools Barracuda [49], CUSHAW [50], SOAP3 [51] and SOAP3-dp have been proposed to the scientific community. Experimental results show that SOAP3, which is the GPU evolution of SOAP2, outperforms the popular tools BWA and Bowtie. When tested to align millions of 100-bp read pairs to the human genome, it resulted at least 7.5 times faster than BWA, and 20 times faster than Bowtie. Moreover, SOAP3 does not exploit heuristics and it is able to align correctly slightly more reads than BWA and Bowtie. SOAP3 is able to align a read to a reference sequence with up to four mismatches while it does not support gapped alignments. Lately, the SOAP3 research team released SOAP3-dp, a new version of the aligner that exploits dynamic programming to support gapped alignments. Compared with BWA, Bowtie2 [52], SeqAlto [53], GEM [54], and the previously mentioned GPU-based aligners, SOAP3-dp is two to tens of times faster, while maintaining the highest sensitivity and lowest false discovery rate on Illumina reads with different lengths. Table 1 summarizes the described tools.

**Table 1:** Short-read mapping tools

Name	Mapping Strategy	Indels Support	Quality evalutation	GPU-based
Barracuda	BWT-based indexing of the reference	Yes	Yes	Yes

BWA	BWT-based indexing of the reference	Yes	Yes	No

Bowtie	BWT-based indexing of the reference	No	Yes	No

CUHSHAW2	BWT-based indexing of the reference	Yes	Yes	Yes

CloudBurst	Hash the reads	Yes	No	No

MAQ	Hash the reads	No	Yes	No

RMAP	Hash the reads	Yes	Yes	No

SHRiMP2	Hash the reads	Yes	Yes	No

SOAP2	BWT-based indexing of the reference	Yes	Yes	No

SOAP3	BWT-based indexing of the reference	No	No	Yes

SOAP3-dp	BWT-based indexing of the reference	Yes	No	Yes

A summary of some of the most popular short-read mapping tools.

### The implemented strategy

**Figure 1:**
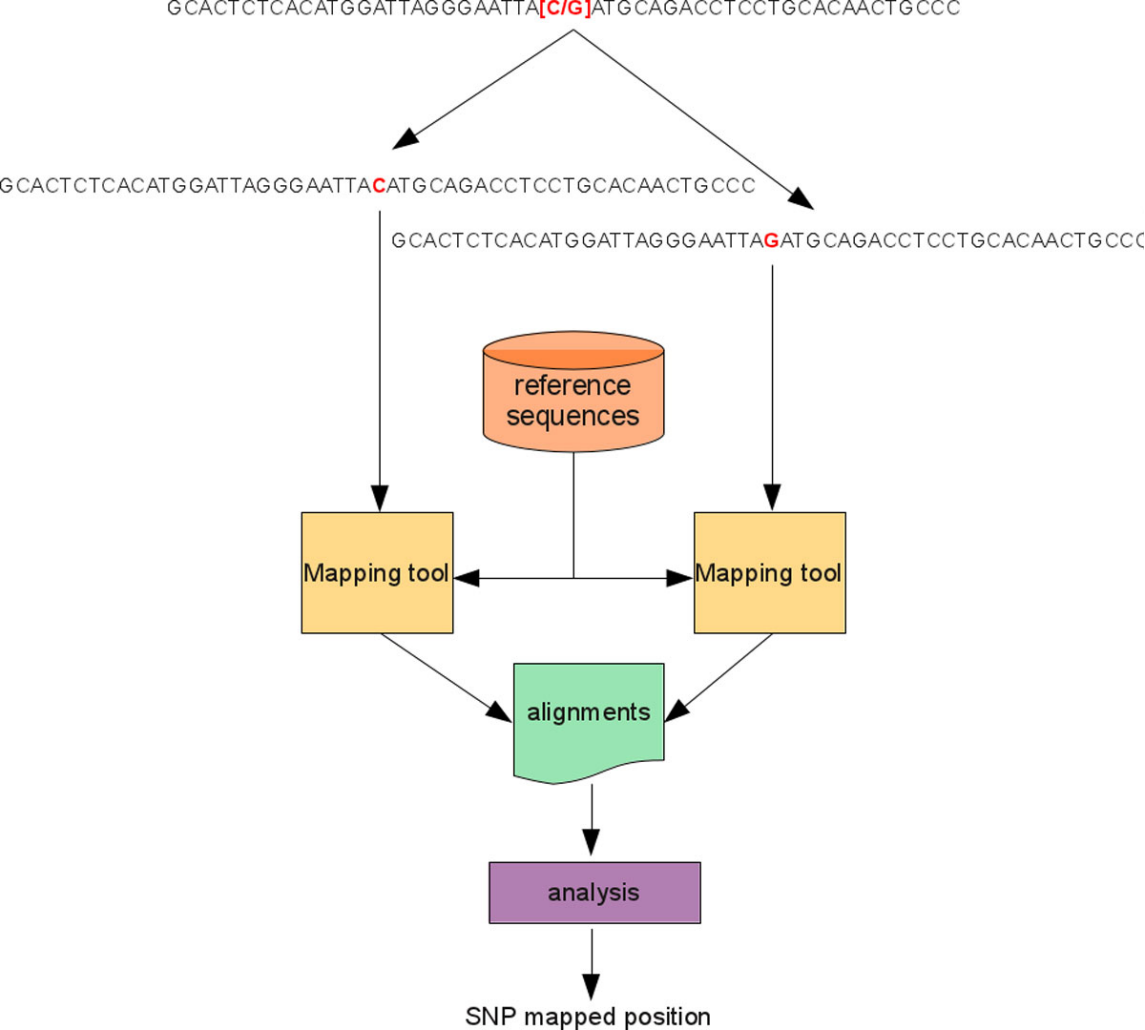
**Using two sequences to represent a SNP**. Two sequences are separately aligned for a SNP. After the alignment, results are analyzed to calculate the absolute position of the SNP.

**Figure 2:**
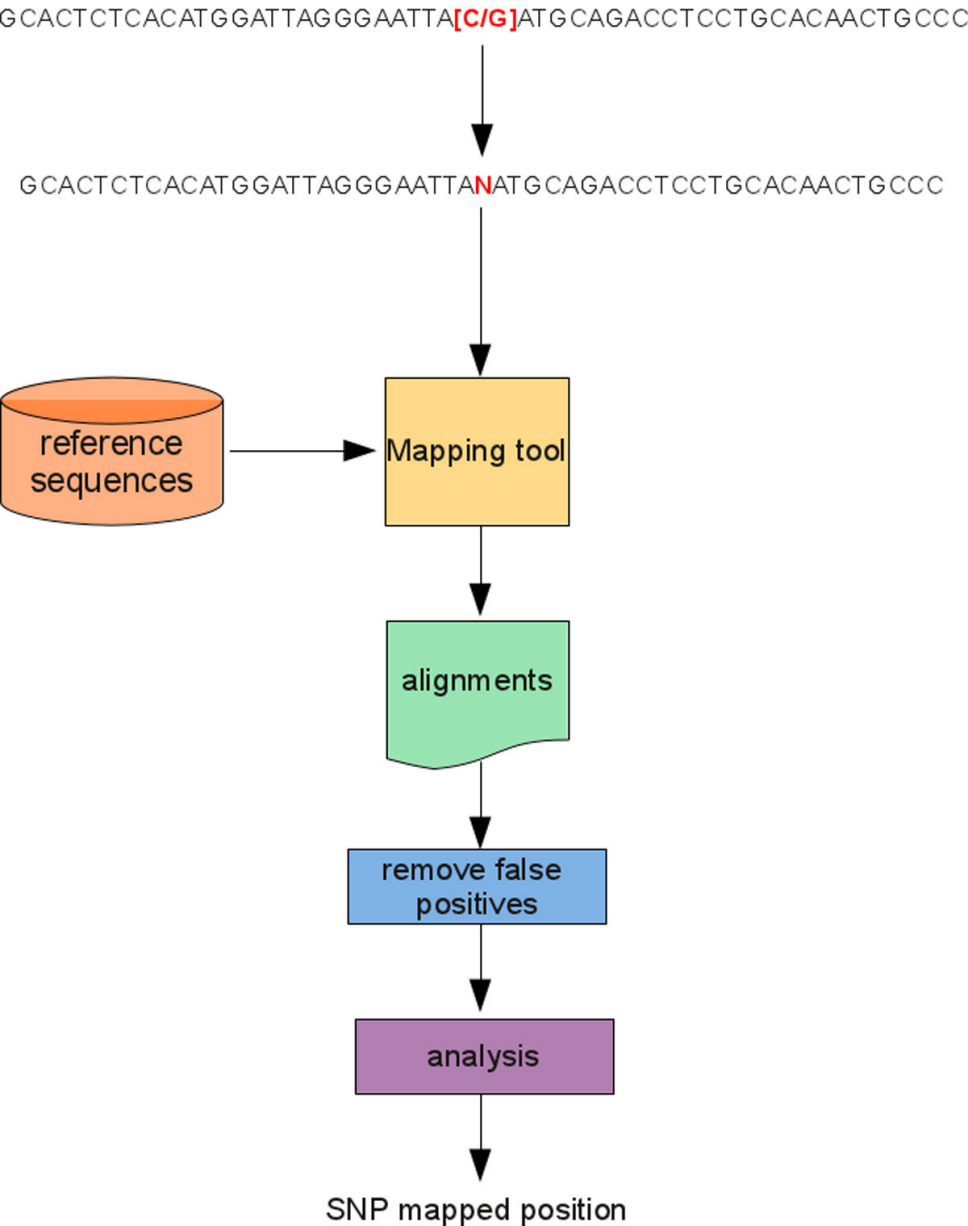
**Using a sequence to represent a SNP**. Only a sequence is aligned for a SNP. After the alignment results are analyzed to remove those false positives and to calculate the absolute position of the SNP.

**Figure 3:**
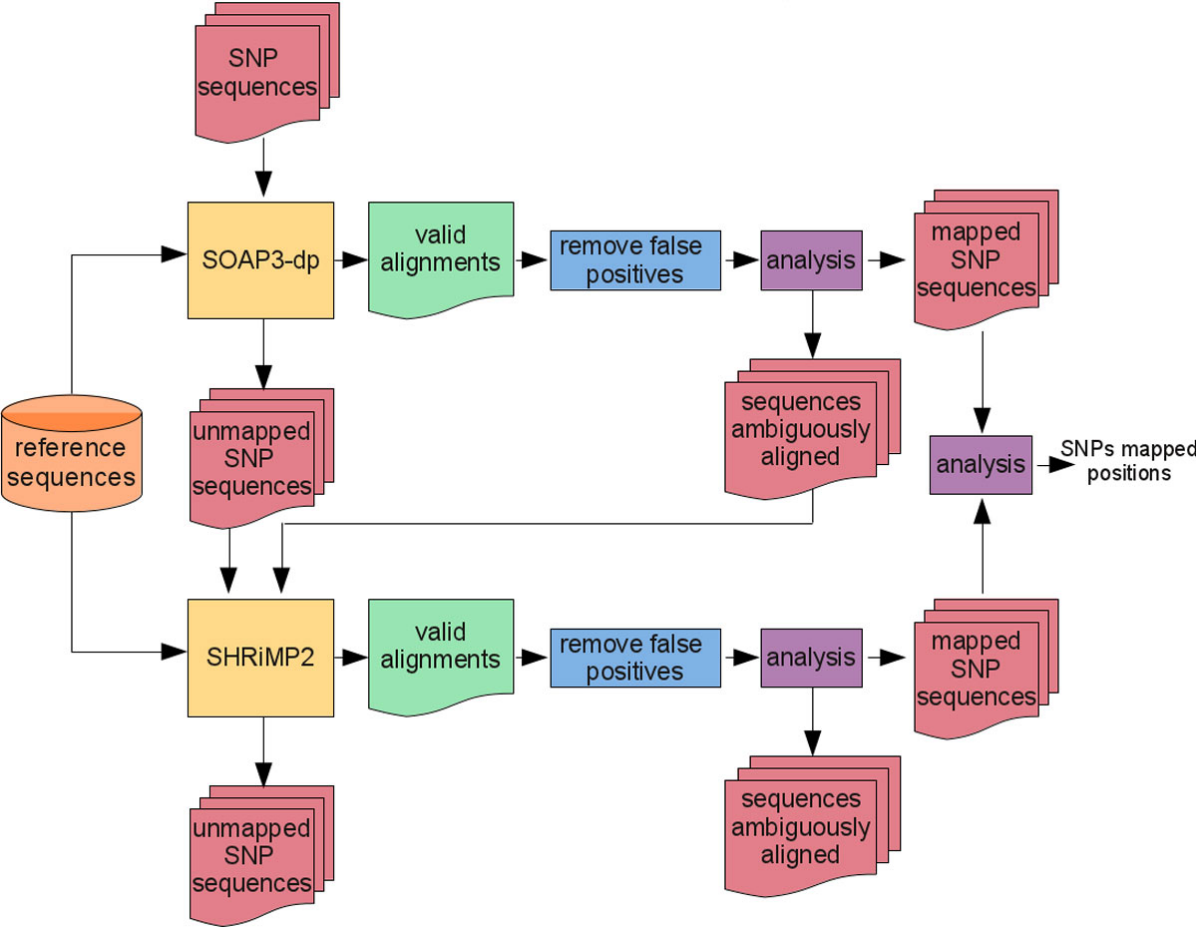
**G-SNPM mapping strategy**. G-SNPM exploits a three-stage pipeline to update the chromosomal position of a SNP. In the first stage, SOAP3-dp is used to unambiguously map a SNP against a reference sequence. Unmapped or ambiguously mapped SNPs are remapped at the second stage by exploiting SHRiMP2. At the third stage, mapped SNP sequences are analyzed to identify the SNP chromosomal position.

As previously seen, a SNP can be synthetically represented by means of a regular expression *R *that uses a single grouping construct to describe the possible alleles. However, short-read mapping tools are not designed to work with sequences described by a regular expression with specialized constructs. Then, two trivial approaches could be used to map a SNP with a short-read mapping tool. As for the former approach (see Figure 1), the probe sequences related to the alleles of a given SNP are dealt with separately in the alignment process. In other words, each probe sequence is aligned against a reference sequence independently from the others using the same mapping tool and identical setting parameters. After that sequences have been aligned, results are merged and analyzed to detect and eventually update the SNPs mapping positions. As for the second approach (see Figure 2), the probe sequences related to the alleles of a given SNP are dealt with simultaneously in the alignment process. To this end, a single sequence must be used to represent the probes related to a SNP. This sequence can be obtained by substituting the grouping construct in *R *that describes the possible alleles with a a*N*y symbol that represents any possible nucleotide. In so doing, the expressiveness of the new sequence increases with respect to that of the starting one, while its information content decreases. In this case, results obtained by aligning the new sequence against a reference sequence must be analyzed to filter out false positive alignments: i.e., those alignments for which the a*N*y symbol that represents the SNP does not match with one of the possible alleles for that SNP. Only after this step alignments can be analyzed to update SNPs mapping positions. This approach can significantly reduce the computational load needed to perform the alignment task. For instance, for biallelic SNPs it will be almost halved with respect to the first approach. Basically, G-SNPM uses this approach to align a sequence representative of a SNP by means an automatic three stage pipeline (see Figure 3).

#### First stage of the pipeline

G-SNPM uses the GPU-based SOAP3-dp short-read mapping tool to align a sequence related to a SNP against its related chromosomal sequence. Typically, a short-read mapping tool is used to map a read against the overall genome. In fact, the genome region from which the read has been generated from the sequencer is unknown. To reduce the running time G-SNPM uniquely aligns each SNP against the reference chromosomal sequence shown in the mapping data of the chip. In fact, it is very unlikely that a SNP has been mapped to a wrong chromosome. Then, since SOAP3-dp exploits the BWT to index a reference sequence, it is necessary to index separately each chromosomal sequence involved in the mapping task.

In general, the alignment process can generate one of three possible results. In particular, also depending on the setting parameters, SOAP3-dp:

i. provides a unique alignment;

ii. provides multiple alignments;

iii. is unable to find an alignment with respect to the given constraints.

As previously explained, the adopted mapping strategy requires that G-SNPM analyzes the resulting alignments to filter out false positives. During the alignment, SOAP3-dp aligns each a*N*y symbol in a sequence as a mismatch against any possible nucleotide in the reference sequence. Therefore, G-SNPM *i*) analyzes each alignment to look for false positives, *ii*) removes them, and then *iii*) updates the edit distance of those alignments classified as true positives. To detect a unique SNP chromosomal position, a unique alignment must be considered valid. To this end, G-SNPM analyzes all valid alignments of each SNP sequence to detect the best hit and discard the others. Basically, the best hit might be detected by calculating the score alignment of each hit and selecting the best. However, G-SNPM analyzes a more complex score. In particular, it detects the best hit by analyzing the BWA-like MAPQ score provided with the last releases of SOAP3-dp that is intended to indicate confidence of read placement accuracy. This score assigns a Phred-like mapping quality score to each read based on match uniqueness, sequence identity, end-pairing, and inferred insert size.

#### Second stage of the pipeline

It is aimed at refining the mapping process. At this stage, G-SNPM tries to remap those SNPs (if any) that have not been mapped at the first stage of the pipeline; in other words, those SNPs for which SOAP3-dp has not been able to provide valid alignments for their representative sequence and/or those SNPs for which G-SNPM has not been able to find unambiguous mapping chromosomal positions (i.e., SNPs for which SOAP3-dp found multiple valid alignments with the same mapping quality score). G-SNPM uses the Smith-Waterman based short-read mapping tool SHRiMP2 to perform this stage of the pipeline. As for the first stage, also in this stage G-SNPM adopts an identical policy to detect and discard false positives alignments that might be found by SHRiMP2, while exploiting the SHRiMP2 mapping quality score to detect the best alignment. At the end of this stage, G-SNPM reports those SNPs for which SHRiMP2 has been unable to find a unique valid alignment of their representative sequences or an unambiguous SNP chromosomal position.

#### Third stage of the pipeline

G-SNPM analyzes unique valid alignments of each successful mapped SNP to calculate the absolute position of each SNP. An output file is generated, containing for each SNP, its name, the related chromosome, the original SNP position, and the mapped SNP position. Moreover, information about the alignment as the strand, and the CIGAR string are also provided. Then, the pipeline is re-executed to map against the overall genome *i*) those SNPs that G-SNPM has been unable to map against a unique chromosomal sequence and *ii*) those SNPs unmapped by the chip vendor.

In G-SNPM reference DNA sequences are accepted in standard FASTA format, whereas SNPs must be represented by using two files: a FASTA file with the representative reads of the SNPs, and another flat file with information about the SNP, in particular the original absolute SNP position and its alleles. Currently, automatic generation of these files is provided for SNP probes of the Illumina Chip. G-SNPM analyzes Illumina files to automatically generate the previously described files for each chromosome.

### Alignment constraints

G-SNPM defines different mapping constraints at the first and second stage of its pipeline, according to the different two mapping tools exploited.

#### First stage

Typically, due the time required to find an alignment, short-read mapping tools allow to set some parameters to limit the maximum alignments allowed for read sequence. For instance, by default Bowtie allows only one alignment for read sequence. In general, this limitation might affect the quality of the final results, especially when no sensitive alignment parameters are imposed. Short-read mapping tools that exploit modern GPUs allow to easily by-pass the limitations of this constraint. By default, SOAP3-dp generates up to 1000 alignments for read. We deem that this is a good constraint and did not modified it in G-SNPM. However, users can easily modify it to decrease, increase, or avoid the upper limit to the alignments that may be found for each sequence.

As already pointed out, SOAP3-dp is the evolution of SOAP3 that exploits dynamic programming to support indels in alignments. Depending on whether dynamic programming is enabled or not, SOAP3-dp will generate gapped or ungapped alignments. When dynamic programming is enabled, SOAP3-dp performs the alignment in two steps. In the first step it looks for ungapped alignments that meet a given constraint on the allowed number of mismatches. Up to 4 mismatches are allowed for this step. In the second step, it exploits dynamic programming to look for gapped alignments. By default, in the first step SOAP3-dp allows up to 2 mismatches to speed-up the overall alignment process. However, G-SNPM modifies this constraint to allow alignments with up 4 mismatches. Users can decreases this value in G-SNPM.

#### Second stage

SHRiMP2 is an accurate short-read mapping tool that has been designed to parallelize the alignment process on multi-core CPUs. By default SHRiMP2 uses only a CPU-core. Then, to speed-up the analysis performed at this stage, G-SNPM assigns all available CPU-cores to SHRiMP2. In particular, it automatically detects the number *N *of available CPU-cores, and then runs SHRiMP2 on *N-1 *cores; a CPU-core is reserved to the operating system. However, it is possible to set manually how many CPU-cores must be assigned to SHRiMP2.

Depending on the number of available CPU cores, it might be useful to limit the maximum number of alignments for sequence, with the aim to reduce the overall mapping time. However, it should be noted that most SNPs are successfully mapped at the first stage of the pipeline. So, the activation of the second stage is sporadic and involves only some SNP sequences. We deemed useful not imposing any limitation on the number of alignments at this stage, to prevent any worsening of the overall accuracy of G-SNPM. At this stage, SHRiMP2 is enabled to allow ungapped alignments. Alignment score and penalties are those of default of SHRiMP2 (i.e., match score = 10; mismatch penalty = 15, gap open penalty = 33, gap extend penalty = 33). It is possible to change these values to meet user constraints.

### Requirements

G-SNPM works on linux based systems with a custom installation of Python (release >=2.7.3) and equipped with a CUDA (Compute Unified Device Architecture) enabled GPU-card. We tested it on two families of NVIDIA GPU cards. In particular tests have been carried out on the NVIDIA FERMI architecture based GTX 480 card, and on the NVIDIA Kepler architecture based k10 and k20c cards. Currently, SOAP3-dp can be run on CUDA-3.2 and CUDA-4.2 releases, while no support for the CUDA 5.0 release has been provided yet. We suggest to scientists interested to use G-SNPM to install the CUDA-4.2 release.

## Results

To assess G-SNPM, we used it in the task to remap about *i*) 1.2 millions of SNPs of the Illumina Chip HumanOmni 1S (version 1) aligned by the chip vendor on the build 37.1 of the human genome, *ii*) 370 thousands of SNPs of the Illumina Chip CNV370 (version 3) aligned on the build 36.1 of the human genome, and *iii*) 318 thousands of SNPs of the Illumina Chip HH300 (version 2) also aligned by the chip vendor on the build 36.1 of the human genome. Experiments have been mainly executed *i*) to highlight discrepancies in respect in map positions provided by the chip vendor, and *ii*) to assess the capability of G-SNPM to deal with the mapping problem. In the following of this section, we first briefly summarize both the hardware configuration and the short-read mapping tool releases exploited to carry out experiments. Then, we describe the way data have been prepared, so that a scientist can easily reproduce experiments. Finally, we present and discuss results.

### Hardware and software configuration

Experiments described hereinafter have been carried out on a 12 cores Intel Xeon CPU E5-2667 2.90GHz with 128 GB of RAM. An NVIDIA Kepler architecture based Tesla k20c card with 0.71 GHz clock rate and equipped with 4.8 GB of global memory has been exploited to execute SOAP3-dp. Moreover, we used the following software releases: SOAP3-dp rel. 2.3.116 and SHRiMP2 rel. 2.2.3.

### Data preparation

We downloaded the *.csv *file version of the Manifest of the analyzed chips from the Illumina website. Then, we used our Illumina parser, which is distributed together with G-SNPM, to automatically generate the working files used by G-SNPM. Successively, we downloaded the builds 36.1, 37.1 and 37.3 of the human genome from the NCBI Reference Sequence Database [55]. Then, we used G-SNPM-Builder (also distributed along G-SNPM), to build the BWT indexes required in the first stage of the pipeline.

### Analysis of mapped SNPs

We used G-SNPM to perform two different experiments. As for the former, we used it to remap the SNPs of each chip against the same genome build previously used by the chip vendor. This experiment permits to put into evidence and to analyze possible discrepancies between the SNPs positions obtained with G-SNPM and those provided by the chip vendor. As for the second experiment, we first used G-SNPM to remap the SNPs against the newest build 37.3 of the human genome and then, we analyzed the reliability of the updated positions. Table 2 reports some details about the SNPs of the analyzed chips. As for the HumanOmni 1S chip, we observed that the vendor provided the positions of 1.180.662 SNPs. As the overall number of SNPs was 1.185.976 no information about the position of 5.314 SNPs was provided. The chip vendor provided the positions of all the 373.397 SNPs of the CNV370 chip, version 3, and of all the 318.237 SNPs of the HH300 chip, version 2.

**Table 2:** Analyzed chips

CHIP name	hg build	SNPs	unmapped SNPs
HumanOmni 1S	37.1	1.185.976	5.314

CNV370 ver 3	36.1	373.397	0

HH300 ver 2	36.1	318.237	0

The first column reports the name of the chips and the second the reference build of the human genome used by the chip vendor to map the SNPs. The third and fourth column report the overall number of SNPs of the chip and the number of them unmapped by the chip vendor, respectively.

### Remapping SNPs against the same reference sequence used by the chip vendor

Table 3 summarizes results obtained remapping SNPs with G-SNPM against the same reference sequences used by the chip vendor. In the table are reported: *i*) the overall number of SNPs mapped using G-SNPM, *ii*) the number of those uniquely mapped, *iii*) the number of SNPs for which G-SNPM has been unable to find any alignment, and *iv*) the number of SNPs for which our tool found positions that differ from those provided by the chip vendor. As for the chip HumanOmni 1S, G-SNPM has been able to remap 4.460 of the 5.314 SNPs for which the chip vendor did not provide any mapping position. Most of these SNPs have been mapped at the first stage of G-SNPM. In particular, they have been mapped by SOAP3-dp looking for ungapped alignments and without exploit any heuristic. Only 35 of these SNPs have been mapped looking for gapped SNPs. In the last column of Table 3 is reported that 4.626 SNPs have been differently mapped with G-SNPM. It should be observed that this value includes also the 4.460 SNPs mapped only by G-SNPM. Analyzing the SNPs mapped by the chip vendor, only 166 of them have been mapped differently with G-SNPM, one on a different chromosome. As for the other chips, G-SNPM mapped uniquely against their related reference build almost all SNPs. Experimental results shown that G-SNPM mapped differently 14.391 SNPs (7 on a different chromosome) of the chip CNV370, version 3, and 1.822 SNPs (none on a different chromosome) of the chip HH300, version 2. Also for these chips G-SNPM mapped almost all SNPs without considering gapped alignments. In our opinion, the differences between the SNPs mapped by G-SNPM with respect those mapped by the chip vendor can be attributed to differences in the alignment algorithms and settings. As reported in the background section, different works have proved that often unreliable positions are provided along the chip, typically due to the fact that not very accurate alignment were obtained. We do not known which algorithm and alignment settings used the vendor. Then, it was difficult to compare the accuracy of our tool with the one of the vendor. In any case we claim that G-SNPM is very accurate. Being based on SOAP3-dp, it looks for ungapped alignments with up to four mismatches without exploiting any heuristics. It is worth pointing out that only a very low percentage of SNPs positions have been calculated starting from gapped alignments and that almost all sequences representative of the SNPs have been uniquely mapped. As for the SNPs of the HumanOmni 1S mapped by G-SNPM and for which the chip vendor did not provide any position, we can suppose that either no valid alignment have been found for them or, conversely, that multiple valid alignments have been found making impossible to unambiguously map these SNPs. As for the 854 SNPs unmapped also by our tool, we assume that G-SNPM tried to map them using some heuristics that did not permitted to find valid alignments.

**Table 3:** Results obtained using G-SNPM to remap the SNPs against the same reference build used by the chip vendor

		SNPs			
**CHIP name**	**hg build**	**mapped**	**uniquely mapped**	**unmapped**	**differently mapped**

HumanOmni 1S	37.1	1.185.122	1.185.118	854	4.626

CNV370 ver 3	36.1	373.397	373.382	0	14.391

HH300 ver 2	36.1	318.237	318.237	0	1.822

A summarization of the discrepancies observed remapping the SNPs with G-SNPM against the same reference builds previously used by the chip vendor to detect the SNPs positions. The first and the second column report the name of the chip and its reference build, respectively. The third column reports the overall number of SNPs mapped using G-SNPM, whereas the fourth column reports the number of them that are uniquely mapped. The fifth column reports the number of SNPs for which G-SNPM did not provide any valid alignment. Finally, the sixth column reports the number of mapped SNPs for which G-SNPM provided different positions with respect to those detected by the chip vendor.

### Remapping SNPs against the build 37.3 of the human genome

Table 4 summarizes results obtained remapping SNPs with G-SNPM against the build 37.3 of the human genome. It should be observed that results are slightly different from those obtained remapping the SNPs against the same build used by the chip vendor. Results show that G-SNPM has been unable to remap some SNPs previously mapped against the oldest builds. As for the chip HumanOmni 1S, almost all SNPs unmapped by the chip vendor have also been mapped against the newest build of the genome. In particular, G-SNPM has been unable to find a valid alignment for 868 SNPs (i.e., 14 SNPs more than in the previous experiment). For the other SNPs unmapped by the vendor, G-SNPM found that they map to the same positions in both builds. As for the other chips, G-SNPM has been unable to find a valid alignment for 23 SNPs of the chip CNV370, version 3, and for 20 SNPs of the chip HH300, version 2. As for the unmapped SNPs, it is possible that, *i*) due to the refinement of the reference sequence, some SNPs are no longer present in the latest build or that *ii*) the refinement of the reference sequence required complex gapped alignments that G-SNPM is unable to find, due to the procedures adopted in the two stages of its pipeline. As in the previous experiment, almost all SNPs have been mapped at the first stage of G-SNPM, while looking for ungapped alignments.

**Table 4:** Results obtained using G-SNPM to remap the SNPs against the build 37.3 of the human genome

CHIP name	hg build	mapped SNPs	uniquely mapped SNPs	unmapped SNPs
HumanOmni 1S	37.3	1.185.108	1.185.103	868

CNV370 ver 3	37.3	373.374	373.371	23

HH300 ver 2	37.3	318.217	318.216	20

The first and the second columns report the name of the chip and its reference build, respectively. The third column reports the overall number of SNPs mapped using G-SNPM, whereas the fourth column reports the number of them uniquely mapped. The fifth column reports the number of SNPs for which G-SNPM did not provide a valid alignment.

To analyze the reliability of our tool, we compared the SNPs positions on the build 37.3 obtained with G-SNPM with *i*) those obtained using a genome remapping tool, and with *ii*) those retrieved by dbSNP. As for the first comparison, we used the NCBI Genome Remapping Service because at the time of writing of the manuscript it is the only assembly-assembly converter tool able to project features from the build 36.1 to the build 37.3, whereas neither the NCBI Genome Remapping Service nor the UCSC LiftOver and Ensembl AssemblyConverter services are currently able to project features from the build 37.1 to the build 37.3. Therefore, this experiment has not been performed for the chip HumanOmni 1S. The NCBI Genome Remapping Service projects the coordinates of a chromosomal region between two different builds of a genome. In this case, we are interested to project against the build 37.3 the coordinates of those regions that contain the SNPs in the build 36.1. Assuming that the SNPs positions provided by the chip vendors are correct, we can identify these regions retrieving the sequences representative of the SNPs, their relative positions within these sequences, and their absolute positions within the chromosome sequence. This information is present in *.csv *files of the Manifest of the chips analyzed for this study. Table 5 summarizes results obtained with the NCBI service. It should be observed that it has been unable to convert the coordinates of several regions if compared with the number of SNPs unmapped by G-SNPM. In particular, it has been unable to project the coordinates of 212 SNPs of the CNV370 chip, version 3, and the coordinates of 28 SNPs of the HH300 chip, version 2. Typically, regions are unmapped either as they are deleted in the new reference or as intersects multiple chains. Moreover, we analyzed if the SNPs mapped with G-SNPM fall in the regions that have been projected with the NCBI service. Results reported in Table 6, show that G-SNPM mapped 7.296 SNPs of the chip CNV370, version 3, in different regions of those obtained with the NCBI service, as well as 454 SNPs of the chip HH300, version 2. Differences might be related to the fact that G-SNPM looks for the nucleotide present in the SNP position and discard those alignments that do not match with one of the possible alleles for the SNP. As the NCBI service does not perform this check, it can report also wrong regions. As for the second comparison, we differently analyzed the SNPs of the HumanOmni 1S chip from those of the chips CNV370 and HH300. In particular, we retrieved from dbSNP the SNPs of the HumanOmni 1S chip unmapped by the vendor. Only 47 of them have a rsID whereas the others have been derived from the 1000 Genomes Project (kgp identifiers). We converted the SNPs with kgp identifiers to rsIDs in dbSNP132 using MegaBLAST [56] to align against the database the sequences representative of the SNPs. We observed that only 859 of 5.314 SNPs were present in dbSNP132 and all of them with multiple positions. Only a little percentage of them validated. For about half of these KGP SNPs, and for all SNPs in the chips with rsID we found in dbSNP the same positions obtained with our tool. As for the other chips, we looked for all SNPs mapped by G-SNPM on dbSNP. About 281 thousands SNPs of the CNV370 chip and about 238 thousands SNPs of the HH300 chip were present in dbSNP. We observed that G-SNPM did not provide identical SNPs positions for 1.447 SNPs of the CNV370 chip and for 1.281 SNPs of the HH300 chip. As for the SNPs for which G-SNPM provided different positions, we observed that dbSNP reports longer flanking sequences that those reported by the vendor. This can be related to the different mappings of G-SNPM as well as the regions unprojected by the NCBI Genome Remapping Service.

**Table 5:** SNPs chromosomal regions projected with the NCBI Genome Remapping Service against the build 37.3 of the human genome

CHIP name	projected regions	unprojected regions
CNV370 v. 3.0	373.185	212

HH300 v. 2.0	318.209	28

A summarization of the results observed converting from the build 36.1 to the build 37.3 of the human genome the coordinates of the regions containing the SNPs detected by the chip vendor. The first column reports the name of the chip, whereas the second and the third report the number of regions successfully projected against the build 37.3 and the number of regions for which the NCBI service has been unable to provide any conversion, respectively.

**Table 6:** Comparison between G-SNPM and the NCBI Genome Remapping Service

CHIP name	regions differently remapped
CNV370 ver 3	7.296

HH300 ver 2	454

The table shows for each analyzed chip the number of SNPs remapped with G-SNPM against the build 37.3 of the human genome whose positions did not fall inside the regions obtained with the NCBI Genome Remapping Service.

### Performance analysis

Table 7 summarizes the performance of G-SNPM in terms of overall mapped SNPs and running time. Results are reported for all experiments we performed and are distinct according to the mapping option. As previously explained, G-SNPM tries to remap against the overall genome sequence those SNPs that have been unmapped against the same chromosomal sequence detected by the chip vendor. In these cases, analysis at the second stage of G-SNPM can require a very long running time. G-SNPM by default tries to align these SNPs only at the first stage. To force the second stage alignment, users must specify the *"D" *option. In the table, results are summarized for both cases. It should be observed that the running time greatly increases when the *"D" *option is used. Only a small percentage of SNPs is further mapped against the overall genome sequence at the second stage of G-SNPM. The time for mapping the SNPs of chip HH300, version 2, do not change after activating this option *"D"*, as all SNPs are in fact mapped at the first stage. Moreover, the table shows that G-SNPM aligns almost 1.2 million of SNPs of the HumanOmni 1S chip faster than the almost 370 thousands SNPs of the CNV370 chip, version 3, and the almost 318 thousands SNPs of the HH300 chip, version 2. Justification must be sought in the fact that in the HumanOmni 1S chip almost all SNPs are mapped at the first stage of G-SNPM. As for the others, G-SNPM required more time to try to map SNPs at the second stage. Table 8, summarizes the number of sequences that G-SNPM tried to align at the second stage of the pipeline and its related processing time. Results shown in Table 8 highlight the presence of a considerable imbalance with respect to the number of sequences processed at the first stage (for instance considering the HumanOmni 1S chip, G-SNPM processed about 1.2 millions of SNPs against the build 37.1 in 20 minutes, of which 13 minutes to process 17 sequences at the second stage).

**Table 7:** Overall analysis of mapped SNPs and running time

		option D disabled		option D enabled	
**CHIP name**	**reference build**	**mapped SNPs**	**global time**	**mapped SNPs**	**global time**

HumanOmni 1S	37.1	1.184.688	20 m	1.185.118	1 h 34 m

HumanOmni 1S	37.3	1.185.031	19 m	1.185.103	1 h 30 m

CNV370 v. 3.0	36.1	373.382	56 m	373.382	2 h 5 m

CNV370 v. 3.0	37.3	373.367	52 m	373.371	2 h 2 m

HH300 v. 2.0	36.1	318.237	29 m	318.237	29 m

HH300 v. 2.0	37.3	318.216	37 m	318.216	37 m

The table is divided in two parts. The first summarizes the performance of G-SNPM when only its first stage has been used to remap against the overall genome sequence those SNPs previously unmapped against the same chromosomal sequence detected by the chip vendor (option "D" disabled). The second part of the table summarizes the performance of G-SNPM when both stages have been used to remap against the overall genome sequence those SNPs previously unmapped against the same chromosomal sequence detected by the chip vendor (option "D" enabled).

**Table 8:** Analysis of the performance at the second stage of G-SNPM

CHIP name	reference build	sequences analyzed	time
HumanOmni 1S	37.1	17	13 m

HumanOmni 1S	37.3	17	12 m

CNV370 v. 3.0	36.1	56	41 m

CNV370 v. 3.0	37.3	81	49 m

HH300 v. 2.0	36.1	10	22 m

HH300 v. 2.0	37.3	36	27 m

A summarization of the performance in terms of running time at the second stage of the G-SNPM. The table shows the number of sequences that G-SNPM tried to align at the second stage and the time required to align them. It is evident a considerable imbalance of the processing time between the first and the second level. The table summarizes the performance with option "D" disabled.

## Conclusions

G-S NPM is a useful and powerful tool that can simplify the work of researchers that plan to remap the SNPs chromosomal positions before to perform any GWAS. Typically, researchers use sequence alignment tools as BLAST or BLAT to update the mapping position of a SNP to a genome or a transcriptome. However, no generalized and/or computationally efficient solutions have been proposed to address this problem. G-SNPM is the only general-purpose tool devised to deal with the mapping of SNPs. Being based on modern GPUs, it exploits the computational power of these hardware accelerators to guarantee a very fast mapping without compromising the accuracy. G-SNPM can be easily integrated in specialized pipelines and workflows devised to cope with specialized GWAS, as well as annotation tasks that requires to remap the SNP probes.

## List of abbreviations used

**BWT**: Burrows-Wheeler Transform; **CUDA**: Compute Unified Device Architecture; **GPU**: Graphics Processing Unit; **GWAS**: Genome Wide Association Studies; **NGS**: Next-Generation Sequencing; **SNP**: Single Nucleotide Polymorphism.

## Competing interests

The authors declare that they have no competing interests.

## Authors' contributions

AM and AO conceived the tool. AM designed and implemented the tool, and drafted the manuscript. AM, AO and GA defined experiments. EM tested the tool during its development. AM, AO and EM analyzed experimental results. AM and GA revised the manuscript. LM coordinated the project, granted access to the computational facilities and maintained the bioinformatics resources. All authors read and approved the final manuscript.

## Declarations

The publication costs for this article were funded by the Italian Ministry of Education and Research (MIUR) through the Flagship (PB05) "InterOmics" project.

This article has been published as part of *BMC Bioinformatics *Volume 15 Supplement 1, 2014: Integrated Bio-Search: Selected Works from the 12th International Workshop on Network Tools and Applications in Biology (NETTAB 2012). The full contents of the supplement are available online at http://www.biomedcentral.com/bmcbioinformatics/supplements/15/S1.
